# Enucleation of a Giant Hemangioma of Liver: Old School Revisited

**DOI:** 10.1155/2015/234767

**Published:** 2015-12-30

**Authors:** Karpagavel ChandraBose, A. Ramanujam, Yega Muthu

**Affiliations:** ^1^Department of General Surgery, Velammal Medical College and Research Institute, Anuppanadi, Madurai, Tamil Nadu 625009, India; ^2^Department of Pathology, Velammal Medical College and Research Institute, Anuppanadi, Madurai, Tamil Nadu 625009, India

## Abstract

Hemangiomas are the most frequent benign hepatic tumours and are usually found in patients aged between 40 and 60 years, more frequently in women. In 30–35% of patients, the lesions are multiple. If the lesions are larger than 4–10 cm, they are coined as “giant” hemangioma. Here, we present a case of giant hemangioma treated with enucleation of the lesion and the advantages of the procedure.

## 1. Introduction

Hemangiomas are the most common benign tumours affecting the liver, with the incidence of 0.4 to 20% [1]. The exact aetiology is still unclear though a genetic predisposition has been proposed [1, 2]. Hemangioma usually occurs in the fifth and sixth decades of life with female preponderance of 5 : 1 [3]. Hemangiomas are composed of multiple, large vessels lined by a single layer of endothelial cells within a thin fibrous stroma. Hemangioma occurs as multiple small lesions which are usually an incidental finding. When the size of the hemangioma exceeds 5 cm, it is termed as “giant” hemangioma. In patients with giant liver hemangioma, observation is justified in the absence of symptoms. Surgical resection is indicated in patients with abdominal (mechanical) complaints, complications, when diagnosis remains inconclusive, rupture, and Kasabach-Merritt syndrome [4]. Here, we present a case of symptomatic giant hemangioma treated with enucleation.

## 2. Case Report

A 40-year-old female with no medical comorbidities presented with complaints of abdominal pain confined to right hypochondria, decreased appetite with early satiety associated with bloating of abdomen after meals. Her family history was unremarkable and she denied any substance abuse. She had similar complaints in the past and was treated symptomatically. She had past history of appendicectomy eight years before the current admission.

**Figure 1:**
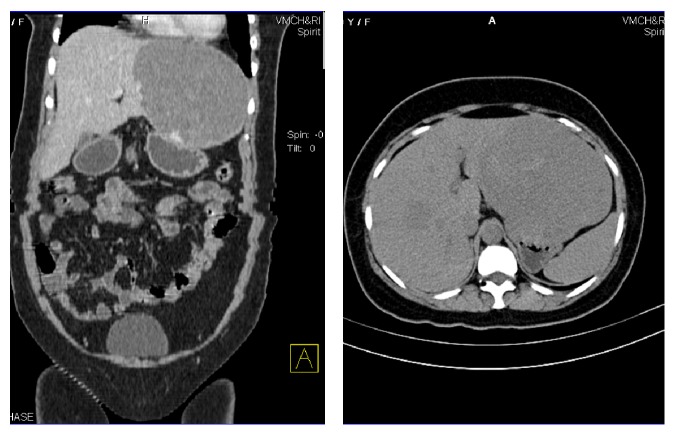
CECT showing multiple hemangioma.

Routine investigations which involved complete blood count, urine analysis, and liver function test were within normal limits (Table 1). With the blood investigations offering no clue, we opted for radiology imaging. Patient underwent a contrast enhanced computed tomography of the abdomen (CECT) and was diagnosed to have multiple hemangioma of varying sizes in both lobes. The largest exophytic lesion was present in the left lobe of liver causing mass effect over the stomach (Figure 1). As the patient was symptomatic, surgical intervention was decided. The choice between enucleation and resection was a debate and finally enucleation was the procedure of choice owing to its less postoperative complications and recurrence rates.

**Figure 2:**
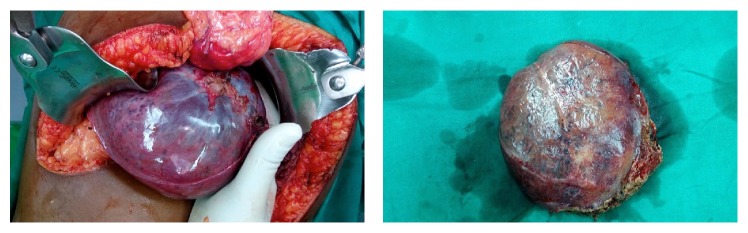
Intraoperative picture of hemangioma.

**Figure 3:**
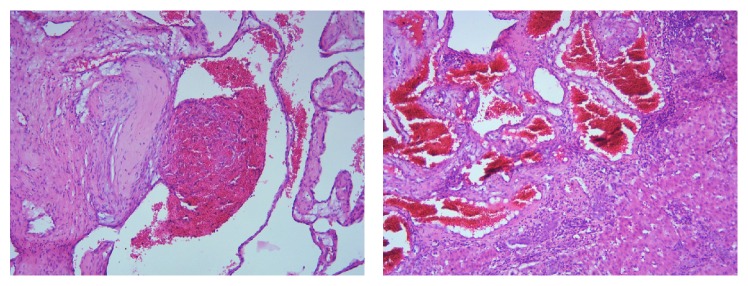
Large vascular channels separated by fibrous strands lined by single layer of flattened epithelial cells with normal mitotic activity.

Patient underwent enucleation of the lesion under general anaesthesia. Bilateral subcostal incisions were made, wound was opened in layers, and mass was excised in toto measuring 20 × 30 cms approximately (Figure 2). After achieving complete haemostasis, wound was closed in layers. The excised mass was sent to histopathology which showed large vascular channels separated by fibrous strands lined by single layer of flattened epithelial cells with normal mitotic activity suggestive of a cavernous hemangioma (Figure 3). Her postoperative period was uneventful. She was observed for a week and then discharged. She is on regular follow-up and is currently asymptomatic.

## 3. Discussion

The management of giant hepatic hemangioma is controversial. Several treatment strategies are available: nonsurgical, surgical treatments (open/laparoscopic) resection or enucleation [4]. Several studies in the past have concluded that clinical observation would suffice in case of a giant hemangioma except for symptomatic patients where surgery is the treatment of choice [4–8]. The new school of techniques including radiotherapy, hepatic artery ligation, or embolization has shown promising results as the reduction in the size and complications [9–11].

The effectiveness of hepatic artery ligation as a treatment for hemangioma has been described anecdotally; however, its benefit is likely transient. Hepatic artery ligation or embolization does play a pivotal role in controlling haemorrhage temporarily from a hemangioma to permit the transfer of a patient to higher centres. Radiotherapy has been used successfully to treat the symptoms and induce involution of hemangioma in few institutions, but the rarity of this occurrence makes the result difficult to interpret. On the whole, the data justifying the use of radiotherapy in hemangioma is scant. However, if surgical therapy is not possible, palliative radiotherapy can be given [12].

Of the various treatment options for giant hemangioma, surgical treatment, including resection or enucleation, provides consistently effective outcome with satisfactory results [13, 14].

The conundrum between enucleation or resection of a giant hepatic hemangioma is dependent on various factors like the certainty of diagnosis, localisation, size and number of lesions, and growth pattern of the hemangioma [4, 13, 14]. Enucleation without a margin of normal liver parenchyma is a justified treatment, since hemangiomas are benign lesions. The advantages of enucleation are less intraoperative blood loss (enucleation: 400 mL versus resection: 1330 mL; *p* = 0.004), less risk of bile leakage (enucleation: 0% versus resection: 8–17%), maximum preservation of functional liver parenchyma, and less overall complications [7, 15–18].

With enucleation, the risk of injury of bile ducts and vessels is minimal, since enucleation is performed just outside of the fibrous capsule surrounding the hemangioma, which is composed of compressed liver parenchyma. Belli et al. showed positive results after enucleation of giant hepatic hemangioma in four patients, with the preservation of sufficient normal liver parenchyma [4, 18]. A comparative study between enucleation and resection by Kuo et al. showed that patient in the enucleation had decreased blood loss [19]. A study by Singh et al. concluded that enucleation is a quicker procedure compared to resection and has less postoperative morbidity [16].

## 4. Conclusion

We report a giant hemangioma successfully treated with enucleation, thus reiterating the advantages of this procedure over resection and other newer modalities.

## Figures and Tables

**Table 1:** Liver profile.

Test description	Observed value	Reference interval
Total bilirubin	**1.08** mg/dL	**0**-**1**
Direct bilirubin	0.36 mg/dL	0–0.3
Indirect bilirubin	0.72 mg/dL	0.2–1
Total protein	6.0 g/dL	6–8
Albumin	3.1 g/dL	3.5–5.5
Globulin	2.9 g/dL	2.5–3.5
AST	44 IU/L	5–40
ALT	36 IU/L	5–40
ALP	210 IU/L	
